# School wellbeing among children in grades 1 - 10

**DOI:** 10.1186/1471-2458-10-526

**Published:** 2010-09-01

**Authors:** Audhild Løhre, Stian Lydersen, Lars J Vatten

**Affiliations:** 1 Department of Public Health, Faculty of Medicine, Norwegian University of Science and Technology, Trondheim, Norway; 2 Unit for Applied Clinical Research, Department of Cancer Research and Molecular Medicine, Faculty of Medicine, Norwegian University of Science and Technology, Trondheim, Norway

## Abstract

**Background:**

Determinants of children's school wellbeing have not been extensively studied. In this cross-sectional study of school children we assessed how factors assumed to promote wellbeing and factors assumed to adversely influence wellbeing were associated with self-reported wellbeing in school.

**Methods:**

Children from five schools, 230 boys and 189 girls in grades 1-10, responded to the same set of questions. We used proportional odds logistic regression to assess the associations of promoting and restraining factors with school wellbeing.

**Results:**

In a multivariable analysis, degree of school wellbeing in boys was strongly and positively related to enjoying school work (odds ratio, 3.84, 95% CI 2.38 to 6.22) and receiving necessary help (odds ratio, 3.55, 95% CI 2.17 to 5.80) from teachers. In girls, being bothered during lessons was strongly and negatively associated with school wellbeing (odds ratio, 0.43, 95% CI 0.22 to 0.85).

**Conclusions:**

Different factors may determine school wellbeing in boys and girls, but for both genders, factors relevant for lessons may be more important than factors related to recess. Especially in boys, the student-teacher relationship may be of particular importance.

## Background

School wellbeing among children has not been extensively studied, despite substantial efforts to develop relevant indicators related to learning ability, health status and health related behaviour in school settings [1]. Konu and colleagues introduced school wellbeing as a global concept [2], and included questions about social relationships and school work as two essential components [3].

Others have used wellbeing as one of several topics to describe how children experience daily life in school [4]. In a multi-national study by Samdal et al [5] feeling safe, experiencing fair treatment and having supportive teachers were associated with a high level of school satisfaction [5]. Children's connectedness to school [6] has also been linked to good health and good academic achievement [7], and it has been suggested that connectedness could be a useful predictor of social competence, emotional distress, risk of dropping out of school, and involvement in criminal activity [8,9].

In a cross-sectional population study of school children, we have assessed whether school wellbeing is associated with factors that are assumed to promote wellbeing, and factors that may restrain the perception of wellbeing. We hypothesised that the assumed promoting factors would favourably influence wellbeing in school, and that perceived problematic factors would be negatively related to school wellbeing.

## Methods

### Participants and procedure

This study is based on a convenience sample of children from five schools in Møre and Romsdal County, Norway, who participated in a project that was organized by the schools. The headmasters agreed to participate in two cross sectional surveys that were set two years apart. The headmasters' decision was approved by each School's Collaborative Committee (sanctioned by law, and including representatives for teachers, parents and children). In the present study, data were used from the first survey that was carried out from May to June 2002.

Three schools had grades from 1 to 7, and two schools had grades from 1 to 10. Altogether 423 children were invited, and included all children from four of the schools and children in grades 7-10 from the fifth school. The children were between seven and 16 years of age at attendance. One child moved before the data collection started, and three children were on sick leave during the study period. Thus, 419 (99%) children were included in the analyses.

Parents were informed about the survey in the context of a school meeting that indicated the start of the project. Information letters signed by the headmaster and by the principal investigator (AL) were sent to all parents, describing the aims of the survey, and emphasising that participation was voluntary, and that the collected information was confidential. Children/parents who did not want to participate were asked to notify their main teacher or headmaster. In each class, teachers informed the children in greater detail about the survey.

Data were collected using a questionnaire (see Additional files 1 and 2) that was developed by the first author (AL) in close collaboration with school nurses, teachers, and headmasters. In the first step of a pilot study, AL had interviewed more than 30 children in grades 1-7, using the questionnaire as a guide. In a second step, approximately 300 children in grades 1-10 from several schools completed the questionnaire after being supervised by trained teachers. The students reported that the questions were easy to understand, and teachers reported that the questionnaire was easy to use among the youngest as well as among older children. Thus, face validity, construct and content validity of the questionnaire were considered to be good.

The data collection of the present study was administered by school nurses and headmasters. Instead of letting all children fill in the questionnaire themselves, 180 children in grades 1-4, 53 children in grades 5-7, and three children in grades 8-10 were interviewed by trained school nurses who used the questionnaire as a guide. Under the instruction of the school nurse or a trained teacher the remaining 183 children completed the questionnaires themselves during a lesson that was allocated to this task.

### Measures

The questionnaire consisted of a combination of items that are assumed to promote school wellbeing, and items that may be adversely associated with school wellbeing. Responses to the questions were ranked on ordinal scales, with four or five response options. Some of the items that were addressed in the questionnaire are more relevant for experiences during lessons and some items are more relevant in recess. Factors assumed to adversely influence school wellbeing included academic problems, disturbances at work, being bothered during lessons, loneliness and victimization (being bullied). Among variables assumed to promote school wellbeing were enjoyment in doing school work, a feeling of receiving help and assistance when needed, and satisfaction with school work. In addition, supportiveness of friends, peers and teachers was assumed to promote school wellbeing. The given responses should be relevant for the current school year.

The reliability of the questionnaire was tested in another material gathered from children in grades 3, 6, and 9. Of 179 eligible children, the questionnaire was completed by 154 (86%) children two times with three weeks apart. The test-retest reliability for the 49 ordinal questions was acceptable with 82% of the Spearman's rho coefficients ranging between 0.45 and 0.64 (mean rho = 0.55), and all p-values < 0.001. With regard to the 12 variables used in the present study correlations varied from 0.49 to 0.71.

In the questionnaire the following items were addressed, each with the corresponding questions:

#### School wellbeing

One global question; "How do you like it at school?" with four response options; very bad (1), not so good, good, and very good (4).

#### Academic problems

Four questions each linked to a certain subject; "Do you have problems with; reading?", "writing?", "mathematics?" or "foreign language (English)?" and each with four response options; no problems (1), some problems, quite a few problems, and lots of problems (4). The response score assigned to "academic problems" was the highest score (one score only) for any of the four questions.

#### Disturbed work

One question; "At school (in class), do you find the necessary peace to work well?" with five response options recoded 5 to 1 to express increasing degree of disturbance; no, never (5), seldom, sometimes, usually, and yes, always (1).

#### Bothered in class

One question; "In class, are you bothered in some way that makes you feel bad?" with five response options; never (1), seldom, sometimes, about every week, and about every day (5).

#### Loneliness

One question; "Do you ever feel lonely at school?" with five response options; never (1), seldom, sometimes, about every week, and about every day (5).

#### Victimization

Three questions; "During recess, are you bothered in some way that makes you feel bad:", where the three questions were specified as by being "teased?"; by being "hit, kicked or pushed?"; or by being "left out, excluded?" and each with five response options; never (1), seldom, sometimes, about every week, and about every day (5). The response score assigned to "victimization" was the highest score (one score only) for any of the three questions.

#### School work enjoyment

One question; "How much do you like schoolwork?" with five response options; not at all (1), not much, so-so, fine, and very much (5).

#### Necessary academic help

One question; "At school (in class), do you feel that you get all the help that you need?" with five response options; no, never (1), seldom, sometimes, usually, and yes, always (5).

#### School work satisfaction

One question; "At school (in class), how pleased are you with your own work?" with five response options; not at all (1), not much, so-so, fine, and very much (5).

#### Friends

One question; "Do you have good friends at school?" with five response options; none (1), one good friend, 2-3 good friends, 4-5 good friends, and many good friends (5).

#### Supportive peers

One question; "Can you talk to other students if something hurtful or difficult happens to you?" with four response options; no, never (1), maybe, probably, and certainly (4).

#### Supportive teacher

One question; "Can you talk to your class advisor if something hurtful or difficult happens to you?" with four response options; no, never (1), maybe, probably, and certainly (4).

### Ethics

The survey was approved by the statutory School Collaborative Committees, and the collection of data was approved by The Norwegian Data Inspectorate.

### Statistics

We used proportional odds logistic regression [10], with school wellbeing as the dependent variable. School wellbeing was constructed as an ordinal variable with four categories, and applying proportional odds is expected to be more efficient than using binary logistic regression [11,12]. In relation to categories of wellbeing in school, the model assumes that the odds ratios will be identical for each category increase in wellbeing.

First, we included each independent factor separately, with adjustment only for gender and grade. Thereafter, all covariates were included simultaneously in a multivariable model. Similar analyses were also conducted separately for boys and girls, and in the multivariable analysis, we tested interactions between relevant factors and gender. All tests were two-sided, and p-values < 0.05 were considered significant. The statistical analyses were performed in SPSS for Windows (version 15.0 SPSS, Chicago, Illinois).

## Results

Among 419 participating children (230 boys and 189 girls), gender was evenly distributed by school grade (Table 1). Global scores for school wellbeing and scores related to variables that are expected to influence school wellbeing are described in Table 2. On a scale from 1 to 4, with 4 as the best, the median score (interquartile range) for school wellbeing was 3 (3-4). The score results indicated that approximately 92% of the children perceived their school wellbeing as good or very good, whereas 8% reported their school wellbeing as bad or not so good. Most of the factors that are expected to influence school wellbeing displayed a similar distribution with a vast majority of the children reporting the two best scores. Only a small proportion of children reported a high degree of perceived problems in lessons and during recess.

**Table 1:** Study participants according to school grade

	Boys		Girls		Total
		
Grade	N	%	N	%	N
1	19	59	13	41	32
2	23	44	29	56	52
3	23	45	28	55	51
4	30	67	15	33	45
5	24	59	17	41	41
6	32	76	10	24	42
7	22	48	24	52	46
8	21	57	16	43	37
9	11	39	17	61	28
10	25	56	20	44	45

Total	230	55	189	45	419

**Table 2:** Distribution of response options for school wellbeing and each of the independent variables

	Response options							
				
	1	2	3	4	5	Total		
Variables	%	%	%	%	%	N	Median	IQR*
School wellbeing ^a^	2.7	5.6	52.9	38.9		414	3	3-4
Academic problems ^b^	26.3	55.4	13.6	4.8		419	2	1-2
Disturbed work ^c^	19.2	39.3	29.5	9.4	2.6	417	2	2-3
Bothered in class ^c^	84.3	7.4	7.6	0.7	0	408	1	1-1
Loneliness ^c^	60.5	21.5	14.8	1.4	1.7	418	1	1-2
Victimization^c^	55.2	24.2	16.5	2.2	1.9	417	1	1-2
School work enjoyment ^d^	2.6	4.8	48.4	35.6	8.6	419	3	3-4
Necessary academic help^d^	1.0	3.4	11.8	43.2	40.6	414	4	4-5
School work satisfaction ^d^	1.4	3.3	32.5	46.7	16.0	418	4	3-4
Friends ^d^	0.2	2.6	15.8	19.4	62.0	418	5	4-5
Supportive peers ^a^	17.5	25.3	15.2	42.0		388	3	2-4
Supportive teacher ^a^	17.0	21.2	18.6	43.2		377	3	2-4

* 25-75th percentile

^a ^From 1 (worst) to 4 (best)

^b ^From 1 (best) to 4 (worst)

^c ^From 1 (best) to 5 (worst)

^d ^From 1 (worst) to 5 (best)

In proportional odds logistic regression analyses, we assessed the association of each variable with the reported school wellbeing score. The left part of Table 3 shows the association of each variable with school wellbeing, after adjustment for gender and grade. In these analyses, all variables except "supportive peers" and "supportive teacher" were significantly associated with school wellbeing, and the direction of the associations was as expected. Thus, variables indicating problems in lessons and recess were negatively related to school wellbeing, whereas enjoying school work showed a strong positive association with school wellbeing (odds ratio, 3.03, 95% CI 2.30 to 4.00), as did the experience of receiving necessary help from the teacher (odds ratio, 3.08, 95% CI 2.35 to 4.05).

**Table 3:** Proportional odds logistic regression with school wellbeing as dependent variable

	Each covariate adjusted only for gender and grade		All covariates, gender and grade, included in the model	
	
Covariates	Odds ratio Estimate (95% CI)	p-value	Odds ratio Estimate (95% CI)	p-value
*Restraining factors*				
Academic problems	0.52 (0.40 to 0.68)	< 0.001	0.88 (0.63 to 1.24)	0.46
Disturbed work	0.57 (0.46 to 0.70)	< 0.001	0.91 (0.70 to 1.19)	0.51
Bothered in class	0.45 (0.32 to 0.62)	< 0.001	0.79 (0.51 to 1.22)	0.29
Loneliness	0.56 (0.45 to 0.70)	< 0.001	0.93 (0.67 to 1.30)	0.68
Victimization	0.52 (0.42 to 0.64)	< 0.001	0.71 (0.52 to 0.97)	0.03
*Promoting factors*				
School work enjoyment	3.03 (2.30 to 4.00)	< 0.001	2.74 (1.95 to 3.85)	< 0.001
Necessary academic help	3.08 (2.35 to 4.05)	< 0.001	2.23 (1.56 to 3.19)	< 0.001
School work satisfaction	1.89 (1.48 to 2.42)	< 0.001	1.16 (0.85 to 1.59)	0.34
Friends	1.43 (1.14 to 1.80)	0.002	1.02 (0.76 to 1.38)	0.88
Supportive peers	1.16 (0.97 to 1.38)	0.10	1.12 (0.91 to 1.38)	0.28
Supportive teacher	1.14 (0.93 to 1.38)	0.21	0.72 (0.55 to 0.93)	0.01

Covariates are factors assumed to either promote or restrain children's wellbeing in school

In the analysis presented on the right part of Table 3 we assessed the association of each variable with school wellbeing with simultaneous adjustment for all the other covariates listed in the table, in addition to gender and grade. After multivariable adjustment, most of the associations that were apparent in the initial analyses (left side of Table 3), were nearly fully attenuated. However, "school work enjoyment" remained strongly associated with school wellbeing (odds ratio, 2.74, 95% CI 1.95 to 3.85), as did receiving "necessary academic help" (odds ratio, 2.23, 95% CI 1.56 to 3.19). Although the association of "victimization" was attenuated, it remained negatively associated with school wellbeing (odds ratio, 0.71, 95% CI 0.52 to 0.97).

The item "supportive teacher" shifted from being positively associated with school wellbeing in the crude analysis to being negatively associated in the multivariable analysis. This change was explored in additional analyses; Spearman correlations showed that "supportive teacher" was correlated with both "school work enjoyment" and "necessary help" (rho, 0.27, and rho, 0.40, respectively, p-values < 0.001), and we consider the change in the direction of the odds ratio to be a likely artefact that may be explained by co-linearity between variables.

We compared associations across grades, grouping the children into three groups (1-4, 5-7, and 8-10). This resulted in low numbers in some categories, and therefore, low precision in some of the analyses, but the associations were not substantially different compared to the analyses of all children in Table 3.

In the separate analyses of boys and girls (Tables 4 and 5), we found that for boys, the results were quite similar to those described in Table 3. In the multivariable analysis (right part of Table 4), there were strong positive associations of "school work enjoyment" (odds ratio, 3.84, 95% CI 2.38 to 6.22) and receiving "necessary academic help" (odds ratio, 3.55, 95% CI 2.17 to 5.80) with level of school wellbeing. For girls, "bothered in class" was the only variable associated with school wellbeing in the multivariable analysis (odds ratio, 0.43, 95% CI 0.22 to 0.85), showing a clear negative association (right part of Table 5).

**Table 4:** Boys only: Proportional odds logistic regression with school wellbeing as dependent variable

	Each covariate adjusted only for grade		All covariates and grade, included in the model	
	
Covariates	Odds ratio Estimate (95% CI)	p-value	Odds ratio Estimate (95% CI)	p-value
*Restraining factors*				
Academic problems	0.57 (0.40 to 0.80)	0.001	0.94 (0.58 to 1.54)	0.82
Disturbed work	0.53 (0.41 to 0.70)	< 0.001	0.99 (0.70 to 1.42)	0.96
Bothered in class	0.50 (0.32 to 0.80)	0.003	1.22 (0.64 to 2.32)	0.55
Loneliness	0.64 (0.47 to 0.86)	0.004	0.97 (0.59 to 1.58)	0.90
Victimization	0.58 (0.43 to 0.78)	< 0.001	0.65 (0.42 to 1.01)	0.06
*Promoting factors*				
School work enjoyment	4.53 (3.10 to 6.62)	< 0.001	3.84 (2.38 to 6.22)	< 0.001
Necessary academic help	3.99 (2.74 to 5.82)	< 0.001	3.55 (2.17 to 5.80)	< 0.001
School work satisfaction	2.31 (1.66 to 3.19)	< 0.001	1.14 (0.73 to 1.80)	0.57
Friends	1.68 (1.22 to 2.32)	0.002	1.53 (0.99 to 2.37)	0.06
Supportive peers	1.02 (0.82 to 1.27)	0.87	1.04 (0.79 to 1.37)	0.80
Supportive teacher	1.14 (0.88 to 1.49)	0.32	0.55 (0.38 to 0.81)	0.002

Covariates are factors assumed to either promote or restrain children's wellbeing in school

**Table 5:** Girls only: Proportional odds logistic regression with school wellbeing as dependent variable

	Each covariate adjusted only for grade		All covariates and grade, included in the model	
	
Covariates	Odds ratio Estimate (95% CI)	p-value	Odds ratio Estimate (95% CI)	p-value
*Restraining factors*				
Academic problems	0.47 (0.30 to 0.71)	< 0.001	0.84 (0.50 to 1.39)	0.49
Disturbed work	0.62 (0.45 to 0.86)	0.003	0.71 (0.46 to 1.11)	0.13
Bothered in class	0.37 (0.23 to 0.61)	< 0.001	0.43 (0.22 to 0.85)	0.02
Loneliness	0.47 (0.34 to 0.65)	< 0.001	0.74 (0.45 to 1.21)	0.23
Victimization	0.45 (0.32 to 0.62)	< 0.001	0.76 (0.48 to 1.22)	0.26
*Promoting factors*				
School work enjoyment	1.66 (1.08 to 2.56)	0.02	1.60 (0.93 to 2.77)	0.09
Necessary academic help	2.18 (1.46 to 3.26)	< 0.001	0.98 (0.54 to 1.78)	0.95
School work satisfaction	1.42 (0.97 to 2.08)	0.07	1.11 (0.68 to 1.80)	0.69
Friends	1.18 (0.85 to 1.64)	0.34	0.69 (0.44 to 1.07)	0.10
Supportive peers	1.44 (1.08 to 1.93)	0.01	1.31 (0.91 to 1.87)	0.14
Supportive teacher	1.12 (0.83 to 1.50)	0.45	1.00 (0.69 to 1.46)	0.99

Covariates are factors assumed to either promote or restrain children's wellbeing in school

We conducted formal testing of interaction with gender in relation to school wellbeing for the four separate variables that clearly differed between girls and boys. Two of these interactions were statistically significant: "school work enjoyment" (p = 0.01) and "necessary academic help" (p = 0.008), whereas interaction tests for "bothered in class" (p = 0.33) and "supportive teacher" (p = 0.48) were not statistically significant.

## Discussion

In this cross-sectional study among school children, high scores on variables that are assumed to promote school wellbeing were associated with higher degree of wellbeing in school, and high scores on variables thought to be perceived as problematic for the children, were related to lower degree of school wellbeing. For boys, high degree of school wellbeing was strongly linked to their enjoyment in school work and to their experience of receiving necessary help from teachers; and for girls, low degree of school wellbeing was strongly related to feeling bothered in class.

The data of this study are population based; all children in the area attended the same public school system. The schools are located in a rural area with a relatively homogenous culture, and therefore, it is difficult to anticipate to which degree the results can be generalised. The very high attendance is an obvious strength of the study, and despite the relatively wide age range (school grade 1-10), the results did not substantially differ across school grades. By carefully following the questionnaire, the school nurse interviewed the youngest children, whereas older children completed the questionnaire themselves. However, we cannot exclude the possibility that this procedure could have influenced the collected information and introduced systematic differences in results between younger and older children.

In the crude analyses, only adjusting for gender and school grade, most factors showed in the expected direction strong associations related to school wellbeing. Thus, factors that were assumed to promote school wellbeing were positively, and factors that were assumed to restrain school wellbeing, were negatively associated with the wellbeing score. However, in the multivariable analyses, where each covariate was adjusted for all the other factors in the model, most of the crude associations were fully attenuated. Moreover, there were patterns in the results that indicated clear differences between boys and girls. Thus, enjoying school work and receiving necessary help from teachers were particularly important for the school wellbeing of boys, whereas in girls, being bothered by others in class was the only variable that remained significant in the multivariable model, and this factor showed a clear negative association with school wellbeing.

It is worth noticing that factors which appear to be important for school wellbeing are related to the lessons, and less to recess, and directly or indirectly, the children's experience with the teachers seems to be important. The effect of peer experiences that other studies have shown to be harmful, such as being bullied [13] or being lonely [14], were attenuated and not statistically significant after multivariable analyses. Similarly, the effects related to having friends and effects of peer support are other relational factors that were attenuated. However, our findings are in accordance with results of other studies using multivariable analyses. Thus, Samdal et al [5] reported that the influence of adults (teachers) was more important for school satisfaction than the influence of other children, and their results also suggested that being alone or being bullied were less important for school satisfaction [5]. In addition, children's reports of teacher likeability (how nice they think their teachers are) seem to be of high value for their school satisfaction [15,16]. The importance of teacher support has also been underlined in longitudinal studies [17,18].

Generally, younger children [5,15,19,20] and girls [5,15,16,19,20] report higher levels of wellbeing in school, but the gender difference may vary by age [5]. In multivariable analyses, our results showed clear gender differences related to factors associated with school wellbeing, whereas in other studies, only minor gender differences were reported [5].

Konu and colleagues, using 80 variables to model school wellbeing showed few gender-dependent patterns with the exception of issues related to health [21]. Nonetheless, there is some evidence to suggest that girls may experience their teachers as more helpful and friendly than boys tend to do [21], and that girls may be more eager to ask questions related to matters they do not understand [22]. On the other hand, adolescent boys may be more competitive academically than girls, whereas girls tend to be more oriented towards relational aspects [22]. These results may support our findings that school wellbeing among boys may depend on academic teacher support, whereas girls seem particularly vulnerable to relational harm indicated by being bothered in class. Others have reported that the approval of other people may be essential for the wellbeing of adolescent girls [23].

## Conclusions

Our results suggest that determinants of school wellbeing among children may differ by gender, but for both genders, the essential factors appear to be closely related to lessons, and not to recess. The teacher's role is important in the promotion of school wellbeing, and a learning environment without harassment is of critical importance.

## Competing interests

The authors declare that they have no competing interests.

## Authors' contributions

The present cross-sectional study is part of a two year follow-up, planned and administered by AL. All the three authors participated in designing the study. AL and SL did the analyses. AL, SL, and LJV interpreted the data and wrote the paper. All authors have read and approved the final manuscript.

## Pre-publication history

The pre-publication history for this paper can be accessed here:

http://www.biomedcentral.com/1471-2458/10/526/prepub

## Supplementary Material

Additional file 1 **Skoletrivsel Elevskjema**. The Norwegian questionnaire (Skoletrivsel - Elevskjema) used in this study was developed by Audhild Løhre (AL). Click here for file

Additional file 2 **School wellbeing Student questionnaire**. Jean Gaffney Kvendset (JGK) translated the original Norwegian questionnaire to English (School wellbeing - Student questionnaire). A retranslation to Norwegian made by a third person, revealed some minor discrepancies. Together, JGK and AL decided the final formulations. Click here for file
