# Supplementary material for: School wellbeing among children in grades 1 - 10
Source: BMC Public Health. 2010 Sep 1;10:526. doi: 10.1186/1471-2458-10-526 (PMC2941687; doi:10.1186/1471-2458-10-526)
Supplement: Additional file 1 — Skoletrivsel Elevskjema. The Norwegian questionnaire (Skoletrivsel - Elevskjema) used in this study was developed by Audhild Løhre (AL). [file 1471-2458-10-526-S1.PDF]

Elevskjema

Skole: _____________________ Klasse: ____ Løpenr.___
År: ______ V ☐ ☐ jente
H ☐ ☐ gutt

SKOLETRIVSEL

*Vi vil gjerne vite hvordan du trives **dette skoleåret**. Sett kryss for det som passer best for deg.*

Timer og friminutt

1. Hva liker du best av timer og friminutt?

timene	friminutta	liker begge deler godt
☐	☐	☐

2. Hva gjør du i friminutta?

3. Hva synes du er bra med friminutta?

4. Hvordan trives du i friminutta?

svært dårlig	dårlig	sånn passe	godt	kjempegodt
☐	☐	☐	☐	☐

5. Har du gode venner på skolen?

ingen	en god venn	2 - 3 gode venner	4 - 5 gode venner	flere gode venner
☐	☐	☐	☐	☐

6. Gleder du deg til timene?

aldri	sjelden	av og til	som oftest	nesten alltid
☐	☐	☐	☐	☐

Skolefag

7. Hvordan liker du skolearbeidet?

svært dårlig	dårlig	sånn passe	godt	kjempegodt
☐	☐	☐	☐	☐

8. Har du problemer med noen av disse fagene:

	ingen problemer	små problemer	store problemer	svært store problemer
lesing	☐	☐	☐	☐
skriving	☐	☐	☐	☐
matematikk	☐	☐	☐	☐
engelsk	☐	☐	☐	☐
gymnastikk	☐	☐	☐	☐

9. Synes du selv at du får den hjelpa du trenger:

	nei, aldri	sjelden	av og til	som oftest	ja, alltid
på skolen	☐	☐	☐	☐	☐
heime med lekser	☐	☐	☐	☐	☐

10. Får du den arbeidsroa du trenger:

	nei, aldri	sjelden	av og til	som oftest	ja, alltid
på skolen	☐	☐	☐	☐	☐
heime	☐	☐	☐	☐	☐

11. Hvor fornøyd er du med det du gjør:

	svært dårlig	dårlig	sånn passe	godt	kjempegodt
på skolen	☐	☐	☐	☐	☐
med lekser heime	☐	☐	☐	☐	☐

Ensomhet

12. Hva betyr det å være ensom?

13. Ser du elever som virker ensom på skolen?

aldri	sjelden	av og til	omtrent hver uke	omtrent hver dag
☐	☐	☐	☐	☐

14. Hva gjør du vanligvis når en elev du kjenner virker ensom?

Her kan du sette ett eller to kryss:

- ☐ gjør ingenting med det, fordi jeg er opptatt med egne aktiviteter
- ☐ gjør ingenting med det, fordi jeg ikke tør
- ☐ gjør ingenting med det, fordi det ikke er mitt problem
- ☐ går bort og prater med eleven
- ☐ ber eleven bli med sammen med andre

15. Hva synes du andre elever bør gjøre når noen virker ensom?

Her kan du sette ett eller to kryss:

- ☐ ingenting
- ☐ gå bort og prate med eleven
- ☐ be eleven bli med sammen med de andre

16. Hva synes du lærerne bør gjøre når elever virker ensomme?

Fortell:

17. Enn du da, føler *du* deg ensom på skolen?

aldri	sjelden	av og til	omtrent hver uke	omtrent hver dag
☐	☐	☐	☐	☐

Den som **aldri** føler seg ensom, går til spørsmål 19.

18. Hvem kan du søke hjelp hos når du føler deg ensom?

Her kan du sette ett eller flere kryss:

- ☐ klassekamerater
- ☐ eldre elever
- ☐ yngre elever
- ☐ lærere / andre voksne
- ☐ ingen

19. Har du kjent deg ensom *før* dette skoleåret starta:

	aldri	sjelden	av og til	omtrent hver uke	omtrent hver dag
i åra før 1. klasse	☐	☐	☐	☐	☐
tidligere skoleår	☐	☐	☐	☐	☐

Plaging

20. Ser du elever som blir erta og plaga i friminutta?

aldri	sjelden	av og til	omtrent hver uke	omtrent hver dag
☐	☐	☐	☐	☐

21. Hva gjør du vanligvis når en elev blir erta og plaga?

Her kan du sette ett eller flere kryss:

☐ gjør ingenting med det, fordi jeg er opptatt med egne aktiviteter
☐ gjør ingenting med det, fordi jeg ikke tør
☐ gjør ingenting med det, fordi det ikke er mitt problem
☐ ber plagerne om å holde opp
☐ henter voksne
☐ sier fra til lærerne etterpå
☐ sier fra til foreldrene mine

22. Hva synes du andre elever bør gjøre når noen blir erta og plaga?

Her kan du sette ett eller flere kryss:

☐ ingenting
☐ be plagerne om å holde opp
☐ gå å hente voksne
☐ si fra til lærerne etterpå
☐ si fra til foreldrene sine

23. Hva synes du lærerne bør gjøre når elever blir erta og plaga?

Fortell:

24. Blir *du* plaga på en sånn måte at du føler det ubehagelig:

	aldri	sjelden	av og til	omtrent hver uke	omtrent hver dag
**på skoleveien**					
Blir erta	☐	☐	☐	☐	☐
Blir slått, sparka, dytta	☐	☐	☐	☐	☐
Blir utestengt og får ikke være med de andre	☐	☐	☐	☐	☐
**i friminutta**					
Blir erta	☐	☐	☐	☐	☐
Blir slått, sparka, dytta	☐	☐	☐	☐	☐
Blir utestengt og får ikke være med de andre	☐	☐	☐	☐	☐
**blir plaga i timene**	☐	☐	☐	☐	☐

Hvis du blir plaga i timene,
så fortell hvordan:

Den som **aldri** blir plaga, går til spørsmål 28.

25. Hvem er det som erter og plager deg i friminutta?

Her kan du sette ett eller flere kryss:

- ☐ klassekamerater
- ☐ eldre elever
- ☐ yngre elever
- ☐ lærere
- ☐ andre voksne

26. Hvem kommer og hjelper deg når du blir plaga?

Her kan du sette ett eller flere kryss:

- ☐ klassekamerater
- ☐ eldre elever
- ☐ yngre elever
- ☐ lærere
- ☐ andre voksne
- ☐ ingen

27. Hvem kan du selv søke hjelp hos når du blir erta og plaga?

Her kan du sette ett eller flere kryss:

- ☐ klassekamerater
- ☐ eldre elever
- ☐ yngre elever
- ☐ lærere / andre voksne
- ☐ ingen

28. Har du vært erta og plaga *før* dette skoleåret starta:

	aldri	sjelden	av og til	omtrent hver uke	omtrent hver dag
i åra før 1. klasse	☐	☐	☐	☐	☐
tidligere skoleår	☐	☐	☐	☐	☐

29. Hender det at *du plager andre* med vilje:

	aldri	sjelden	av og til	omtrent hver uke	omtrent hver dag
erter	☐	☐	☐	☐	☐
slår, sparker, dytter	☐	☐	☐	☐	☐
holder andre utafor	☐	☐	☐	☐	☐

Trivsel og humør

30. Hvordan har humøret ditt vært den siste tida?

veldig dårlig	ikke så bra	godt	kjempegodt
☐	☐	☐	☐

31. Hvordan har du hatt det den siste tida? Hvor ofte har du:

	aldri	sjelden	av og til	veldig ofte	hele tida
vært glad	☐	☐	☐	☐	☐
vært trist	☐	☐	☐	☐	☐
følt deg trygg	☐	☐	☐	☐	☐
vært engstelig	☐	☐	☐	☐	☐
hatt vondt i magen	☐	☐	☐	☐	☐
hatt vondt i hodet	☐	☐	☐	☐	☐
hatt andre plager	☐	☐	☐	☐	☐

32. Gruer du deg til friminutta?

aldri	sjelden	av og til	som oftest	nesten alltid
☐	☐	☐	☐	☐

33. Gruer du deg til timene?

aldri	sjelden	av og til	som oftest	nesten alltid
☐	☐	☐	☐	☐

34. Hvem kan du snakke med hvis noe vondt eller vanskelig skulle hende :

	nei, aldri	kanskje	sannsynligvis	helt sikkert
andre elever	☐	☐	☐	☐
klassestyrer	☐	☐	☐	☐
andre lærere	☐	☐	☐	☐
foreldrene mine	☐	☐	☐	☐
andre voksne	☐	☐	☐	☐
ingen ☐				

35. Hvordan trives du på skolen?

veldig dårlig	ikke så bra	godt	kjempegodt
☐	☐	☐	☐

36. Fortell hvorfor du trives som du gjør på skolen:

37. Tenk deg et super-fint friminutt. Hva ville du helst holde på med da?
Fortell:
