# Supplementary material for: School wellbeing among children in grades 1 - 10
Source: BMC Public Health. 2010 Sep 1;10:526. doi: 10.1186/1471-2458-10-526 (PMC2941687; doi:10.1186/1471-2458-10-526)
Supplement: Additional file 2 — School wellbeing Student questionnaire. Jean Gaffney Kvendset (JGK) translated the original Norwegian questionnaire to English (School wellbeing - Student questionnaire). A retranslation to Norwegian made by a third person, revealed some minor discrepancies. Together, JGK and AL decided the final formulations. [file 1471-2458-10-526-S2.PDF]

Student questionnaire

School: ___________________________ Grade:___ Number___

Year: ________ spring ☐ autumn ☐ ☐ girl ☐ boy

School wellbeing

We want to know how you feel about this school year. Put an x in the box that best describes you.

Class and recess

1. Which do you like best:

class	recess	like both equally well
☐	☐	☐

2. What do you do during recess?

3. What do you like about recess?

4. How much do you like recess?

not at all	not much	so-so	fine	very much
☐	☐	☐	☐	☐

5. Do you have good friends at school?

none	one good friend	2-3 good friends	4-5 good friends	many good friends
☐	☐	☐	☐	☐

6. Do you look forward to class?

never	seldom	sometimes	usually	almost always
☐	☐	☐	☐	☐

Subjects

7. How much do you like schoolwork?

not at all	not much	so-so	fine	very much
☐	☐	☐	☐	☐

8. Do you have problems with any of these subjects:

	no problems	some problems	quite a few problems	lots of problems
reading	☐	☐	☐	☐
writing	☐	☐	☐	☐
mathematics	☐	☐	☐	☐
foreign language (English)	☐	☐	☐	☐
P.E.	☐	☐	☐	☐

9. Do you feel that you get all the help that you need:

	no, never	seldom	sometimes	usually	yes, always
at school (in class)	☐	☐	☐	☐	☐
with homework	☐	☐	☐	☐	☐

10. Do you find the necessary peace to work well:

	no, never	seldom	sometimes	usually	yes, always
at school (in class)	☐	☐	☐	☐	☐
with homework	☐	☐	☐	☐	☐

11. How pleased are you with your own work:

	not at all	not much	so-so	fine	very much
at school (in class)	☐	☐	☐	☐	☐
with homework	☐	☐	☐	☐	☐

Loneliness

12. What does it mean to be lonely?

13. Do you ever see students at school who seem to be lonely?

never	seldom	sometimes	about every week	about every day
☐	☐	☐	☐	☐

14. What do you usually do if someone you know at school seems lonely?

You may mark one or two boxes:

- ☐ don’t do anything because I’m busy with my own activities
- ☐ don’t do anything because I don’t dare
- ☐ don’t do anything because it’s not my problem
- ☐ go over and talk with the student
- ☐ ask the student to join in with the others

15. What do you think other students should do when they see someone who seems lonely?

You may mark one or two boxes:

- ☐ nothing
- ☐ go over and talk with the student
- ☐ ask the student to join in with the others

16. What do you think teachers should do when students seem to be lonely?

Explain:

17. What about you, do you ever feel lonely at school?

never	seldom	sometimes	about every week	about every day
☐	☐	☐	☐	☐

If you never feel lonely, go to question 19.

18. Who can you ask for help when you feel lonely?

You may mark one or more boxes:

- ☐ classmates
- ☐ older students
- ☐ younger students
- ☐ teachers/other adults
- ☐ no one

19. Did you ever feel lonely before the start of this school year?

	never	seldom	sometimes	about every week	about every day
pre-school years	☐	☐	☐	☐	☐
previous grades	☐	☐	☐	☐	☐

Bullying

20. Do you ever see students who are teased or bothered during recess?

never	seldom	sometimes	about every week	about every day
☐	☐	☐	☐	☐

21. What do you usually do when a student is teased or bothered?

You may mark one or more boxes:

- ☐ don’t do anything because I’m busy with my own activities
- ☐ don’t do anything because I don’t dare
- ☐ don’t do anything because it’s not my problem
- ☐ tell the bullies to stop
- ☐ get adults
- ☐ tell the teachers later
- ☐ tell my parents

22. What do you think other students should do when someone is teased or bothered?

You may mark one or two boxes:

- ☐ nothing
- ☐ tell the bullies to stop
- ☐ get adults
- ☐ tell the teachers later
- ☐ tell their parents

23. What do you think teachers should do when students are teased or bothered?

Explain:

24. Are you bothered in some way that makes you feel bad?

	never	seldom	sometimes	about every week	about every day
**to and from school**					
teased	☐	☐	☐	☐	☐
hit, kicked, pushed	☐	☐	☐	☐	☐
left out, excluded	☐	☐	☐	☐	☐
**during recess**					
teased	☐	☐	☐	☐	☐
hit, kicked, pushed	☐	☐	☐	☐	☐
left out, excluded	☐	☐	☐	☐	☐
**bothered in class**	☐	☐	☐	☐	☐

If you are bothered in class, explain how:

If you are never bothered, go to question 28.

25. Who teases or bothers you during recess?

You may mark one or more boxes:

- ☐ classmates
- ☐ older students
- ☐ younger students
- ☐ teachers
- ☐ other adults

26. Who comes and helps you when someone has bothered you?

You may mark one or more boxes:

- ☐ classmates
- ☐ older students
- ☐ younger students
- ☐ teachers
- ☐ other adults
- ☐ no one

27. Who can you ask for help when you have been teased or bothered?

You may mark one or more boxes:

- ☐ classmates
- ☐ older students
- ☐ younger students
- ☐ teachers/other adults
- ☐ no one

28. Were you ever teased or bothered before the start of this school year?

	never	seldom	sometimes	about every week	about every day
pre-school years	☐	☐	☐	☐	☐
previous grades	☐	☐	☐	☐	☐

29. Do you ever bother others on purpose (intentionally):

	never	seldom	sometimes	about every week	about every day
tease	☐	☐	☐	☐	☐
hit, kick, push	☐	☐	☐	☐	☐
shut out, exclude	☐	☐	☐	☐	☐

Wellbeing and mood

30. What kind of mood have you been in lately?

very bad	not so good	good	very good
☐	☐	☐	☐

31. How have you felt lately? How often have you:

	never	seldom	sometimes	often	always
been happy	☐	☐	☐	☐	☐
been sad	☐	☐	☐	☐	☐
felt safe	☐	☐	☐	☐	☐
felt anxious	☐	☐	☐	☐	☐
had a stomach ache	☐	☐	☐	☐	☐
had a head ache	☐	☐	☐	☐	☐
had other problems	☐	☐	☐	☐	☐

32. Do you dread recess?

never	seldom	sometimes	often	almost always
☐	☐	☐	☐	☐

33. Do you dread classroom time?

never	seldom	sometimes	often	almost always
☐	☐	☐	☐	☐

34. Who can you talk to if something hurtful or difficult happens to you:

	no, never	maybe	probably	certainly
other students	☐	☐	☐	☐
class advisor	☐	☐	☐	☐
other teachers	☐	☐	☐	☐
my parents	☐	☐	☐	☐
other adults	☐	☐	☐	☐

no one ☐

35. How do you like it at school?

very bad	not so good	good	very good
☐	☐	☐	☐

36. Explain why you feel this way about school:

37. Think about a fantastic recess. What would you like to be doing then?

Explain:

English translation by Jean Gaffney Kvendset, June 2010
